# Investigating the presence and detectability of structural peripheral arterial changes in children with well-regulated type 1 diabetes versus healthy controls using ultra-high frequency ultrasound: a single-centre cross-sectional and case-control study

**DOI:** 10.1016/j.eclinm.2025.103097

**Published:** 2025-02-13

**Authors:** Ebba Bergdahl, Gun Forsander, Frida Sundberg, Linda Milkovic, Frida Dangardt

**Affiliations:** a Department of Molecular and Clinical Medicine, Institute of Medicine, Sahlgrenska Academy, University of Gothenburg, Sweden; b Department of Paediatrics, Institute of Clinical Sciences, Sahlgrenska Academy, University of Gothenburg, Gothenburg, Sweden; c Children's Heart Centre, The Queen Silvia Children's Hospital, Sahlgrenska University Hospital, Region Västra Götaland, Gothenburg, Sweden

**Keywords:** Ultra high frequency ultrasound, Cardiovascular prevention, Children, Type 1 diabetes

## Abstract

**Background:**

Children with type 1 diabetes have an increased risk of macrovascular complications. This study used ultra-high frequency ultrasound (UHFUS), enabling differentiation of intima thickness (IT), and media thickness (MT) in peripheral arteries, to examine early peripheral arterial changes in children with type 1 diabetes (CWD).

**Methods:**

This cross-sectional and case-control study performed at the Queen Silvia Children's Hospital, Gothenburg, Sweden included CWD, aged 6–15.99 y/o, diabetes duration ≥5 years, compared to age and sex matched healthy controls. Exclusion criteria included other medical conditions or treatments besides insulin, abnormal examination findings or inability to handle extensive examinations. UHFUS measurements from the radial, dorsal pedal (DP), and carotid arteries as well as blood samples, blood pressure (BP)- and BMI z-score were collected from all study participants, and glucometrics from CWD.

**Findings:**

Study inclusion was performed during 02/25/2019–06/28/2022, and a total of 50 CWD, and 41 healthy controls were included in the study. Of these, five CWD and four healthy controls were excluded, resulting in 45 (22 girls (49%), 23 boys (51%)) CWD (12.0 (2.3) y/o) and 37 (19 girls (51%), 18 boys (49%)) healthy controls (11.3 (2.5) y/o) included in data analysis. CWD had a mean HbA1c of 6.6% (48.1 mmol/mol), higher DBP z-scores (p = 0.019), DP IT, DP intima-media thickness (IMT), and radial IT compared with controls (p = 0.003, p = 0.008, and p = 0.002, respectively). Carotid IT was correlated with time in range (r = −0.47, p = 0.014), time in tight range (r = −0.64, p < 0.001), and glucose variability (r = 0.40, p = 0.004) in CWD. Time in tight range and longitudinal HbA1c were the strongest determinants for carotid IT in CWD, and type 1 diabetes diagnosis was the strongest determinant for IT across all arteries.

**Interpretation:**

Children with well-regulated type 1 diabetes show early vascular changes in radial and DP arteries. Regression analyses indicate significant links between IT and hyperglycaemia and type 1 diabetes diagnosis respectively, indicating that structural arterial changes start in the intima. Our findings further emphasise increased time in normoglycemia as the most crucial action to prevent cardiovascular complications in type 1 diabetes. Additional larger studies are needed to confirm and further interpret the meaning of these results.

**Funding:**

ALF-agreement, Child Diabetes Foundation, 10.13039/501100009804Swedish Diabetes Foundation, and the Sahlgrenska University Hospital Foundations.

**Research in context**

**Evidence before this study**

We searched PubMed during the period 1st of August 2022—31st of May 2024 using the following search terms (vascular OR cardiovascular) AND (children OR pediatric OR paediatric) AND (type 1 diabetes) filtering for humans, including articles from 1990 up to the date of the literature search. Briefly, structural arterial changes have been previously described in both the aorta and the carotid arteries in children with type 1 diabetes (CWD). These changes are associated with glycaemic and metabolic control, and hyperglycaemia is considered the most important cardiovascular (CV) risk-factor in type 1 diabetes, enhanced by traditional CV risk-factors. Studies exploring structural changes to more peripheral arteries by using ultra-high frequency ultrasound (UHFUS) has not yet been conducted in CWD. With an axial resolution of up to 20 μm, UHFUS enables differentiation of the intimal and medial layers of the arterial wall. This allows for detection of smaller, likely earlier vascular changes, as well as more detailed pathophysiological considerations.

**Added value of this study**

CWD in this study show comparably very good glycaemic control (HbA1c of 6.6 ± 0.6% (48.1 ± 6.0 mmol/mol)) and no presence of other CV risk-factors. Regardless, peripheral arterial changes were found, predominately involving the dorsal pedal (DP) artery. To the best of our knowledge this is the first study to show detectible vascular changes in well-regulated paediatric type 1 diabetes. The UHFUS, allowing examination of smaller arteries as well as differentiation between IT and MT, is likely the reason why what are believed to be earlier peripheral arterial changes than previously shown could be detected. The strong associations between carotid IT and glucometrics found in CWD in this study are interesting findings in need of further confirmation suggesting an important connection between hyperglycaemia and early changes to the intima.

**Implications of all the available evidence**

Our results confirm and further emphasise the importance of glycaemic control to prevent CV complications in type 1 diabetes. We find important associations between glycaemic control and IT as well as blood pressure, indicating parallel processes affecting the vasculature by increasing both IT and MT. Our results show detectable vascular changes even in this cohort of children with well-regulated type 1 diabetes. Hyperglycaemia plays a crucial role in complication development, suggesting that striving for normoglycaemia already from type 1 diabetes diagnosis may be one of the most important cardio-preventive actions in paediatric type 1 diabetes. When studying populations of CWD with well-controlled glycaemic levels—such as in Sweden, where 48% of children aged 6–15.99 years with a diabetes duration of at least 5 years maintain an HbA1c below 52 mmol/mol—data is essential to assess their current vascular risk.

## Introduction

Macrovascular complications in type 1 diabetes constitutes of cardio-, cerebro- and peripheral vascular disease.^1^ Hyperglycaemia has been recognised as the most important risk factor for early atherosclerosis in type 1 diabetes,^2^^,^^3^ and in individuals diagnosed before 10 years of age, cardiovascular disease (CVD) may cause a loss of 17.7/14.4 years of life in women/men, respectively.^4^ Even though cardiovascular (CV) complication rate in type 1 diabetes has decreased in areas with access to specialised healthcare,^5^^,^^6^ children with type 1 diabetes (CWD) still show increased prevalence of modifiable cardiovascular risk-factors,^7^ indicating a need for new, more individualised preventive treatment strategies.^7^^,^^8^ The use of advanced diabetes technology is one of the reasons for rapidly improving treatment results in type 1 diabetes. Insulin-pump therapy decreases the risk of CVD compared to treatment with multiple insulin-injections,^9^ and continuous glucose monitoring (CGM) provide several glucometrics mirroring the day-to-day glucose variability.^10^ Insufficient glycaemic control measured by CGM has been associated with both micro- and macrovascular complications in type 1 diabetes.^11^^,^^12^

CVD prognostic factors in type 1 diabetes apart from dysglycemia are increased blood pressure and blood lipid levels as well as microalbuminuria.^13^ Complications accelerate during puberty.^14^ Increased carotid intima-media- thickness (cIMT), a marker for subclinical atherosclerosis, has been found in CWD, associated with poor metabolic control,^15^^,^^16^ diabetes duration, systolic blood pressure (SBP), and body mass index (BMI).^16^^,^^17^

Sensitive methods for early detection of CV impact in type 1 diabetes are important keys to a broader mechanistic understanding of the premature arterio- and atherosclerotic process in type 1 diabetes and a prerequisite for early intervention.

Our objectives were to use ultra-high frequency ultrasound (UHFUS), to investigate the presence and detectability of structural vascular changes in the radial, dorsal pedal (DP) and carotid arteries and, in relation to the collected structural vascular measures, to explore associated markers of glycaemic and metabolic control, in a cohort of children with well-regulated type 1 diabetes compared to age, and sex matched healthy controls in a cross-sectional case control study. Our specific objectives were; first to use UHFUS to differentiate between the intimal and medial layers of the arterial wall for a detailed morphological vascular assessment, enabling the detection of minor structural changes in peripheral arteries. Second, we aimed to explore various markers for glycaemic and metabolic control in relation to the UHFUS morphological vascular assessments.

By employing these sensitive methods, we aim to advance cardiovascular prevention in paediatric type 1 diabetes.

We hypothesised that our sensitive UHFUS examinations will enable detection of small and early vascular changes even in this cohort of children with well-regulated type 1 diabetes, and without the presence of manifest traditional cardiovascular (CV) risk factors, compared to healthy controls. We further hypothesised that these vascular changes could be associated with analysed glycaemic and metabolic markers.

## Methods

### Study design

This was a single centre, cross sectional, case control study, performed at the Queen Silvia Children's hospital in Gothenburg, Sweden, currently monitoring approximately 650 CWD. The study was approved by the local ethic's board, Gothenburg 2018-10-12 (Ref: 622-18) and was conducted according to the declaration of Helsinki. Presentation of study results is performed in accordance with the STROBE reporting guidelines for cross sectional studies. Participation was voluntary and written consent was collected from all study participants and their caregivers, and study inclusion was performed during 02/25/2019–06/28/2022.

Our inclusion criteria were type 1 diabetes duration ≥5 years and age 6–15.99 y/o, and our exclusion criteria were other medical treatments or conditions such as medical treatment for ADHD, asthma or coeliac disease, pathological examination findings, such as hypertension or obesity, and/or inability to cope with the extensive examination protocol. CWD and caregivers were asked by the paediatric diabetologist or nurse to participate in the study and healthy controls were matched on group-level by age and sex, and recruited via the type 1 diabetes participants, i.e., siblings and friends, as well as through announcements in the local area and on social media.

Scanning of the radial, dorsal pedal (DP), and carotid arteries using UHFUS was performed, and blood- and urine samples were collected from all study participants. CGM data from the last 14 days was available and collected from 31 of the CWD who used a Dexcom G5 or G6 CGM-device with a functioning connection to the online CGM analysing tool, Diasend, where daily glucose statistics are presented for the CGM user and his/her caregivers, physician and/or nurse. HbA1c values from CWD, starting three months after type 1 diabetes diagnosis and every 3rd month until the study examination, was collected from the Swedish National Diabetes Registry (NDR) and a mean value was calculated (Longitudinal HbA1c).

### Primary and secondary outcomes

Primary outcomes were the vascular wall-measures; intima thickness (IT), media thickness (MT), and intima-media thickness (IMT), from the radial, DP, and carotid arteries. Secondary outcomes: analysed glycaemic and metabolic markers associated with the vascular wall-measures, IT, MT, and IMT of the examined arteries.

### Ultra-high frequency ultrasound (UHFUS)

UHFUS (Vevo MD, Visualsonics, Canada) was used for scanning the left radial and DP arteries and both carotid arteries. Examinations were performed with the patient in a supine position, using a linear probe (Range: 29–71 MHz) with an axial resolution of up to 20 μm. In previously published material from the same research group, inter-observer variability has been examined in a subgroup of 10 children with a mean coefficient of variation of 4 ± 1%.^18^ Carotid ultrasound was performed approximately one cm below the carotid bulb, the radial artery was examined 1–2 cm proximal to the skinfold separating the palma manus from the regio antebrachi anterior and the DP was examined distal to the most prominence of the navicular bone, as earlier described in closer detail.^19^ Recordings of digital cine-loops of 3 consecutive cardiac cycles were collected in B-mode for each artery. Workspace Vevo LAB, Visualsonics (Version 3.1.1) was used for vascular measurements, which were performed in an enlarging window using callipers, when vascular diameter was at its largest. Diameter, IT and MT was measured in three different cardiac cycles, and IMT was calculated as the sum of IT and MT. An average of three separate measurements were calculated. Vascular diameter was defined as the distance between the edge of the intima-media interface of the near wall and the edge of the far wall intima-lumen interface. IT and MT were measured on the far wall, IT was defined as the distance between the intima-lumen interface and the intima-media interface and MT was defined as the distance between the intima-media interface and the media-adventitia interface.

### Demographic data

Height was measured with a stadiometer to the nearest 0.1 cm and weight was measured on a digital scale to the nearest 0.1 kg. Waist and hip circumference were measured with the patient in a standing position with the feet hip wide, at the level of the navel and of the largest part of the glutaeal region, respectively. BMI z-score was calculated using the Centre for Disease Control (CDC) growth charts.^20^ Office blood pressure (BP) was measured using an automated BP monitor (Medi-Dyne) in the right arm after 20 min rest in a supine position. An average of three measurements was used.

### Biochemistry

Venous blood- and urine samples were collected from all study participants. Non fasting serum lipid profile, triglycerides, total cholesterol, high-density lipoprotein (HDL) and low-density lipoprotein (LDL), blood glucose, HbA1c, white blood cell count (WBC), and Cystatin C were analysed. All blood samples were measured photometrically using an enzymatic method, at an accredited lab.

### Statistical analysis

With 23 patients in each group, the probability was 91 percent that the study could detect a difference of 0.030 mm in medial thickness measured by UHFUS in the peripheral arteries, at a two-sided 0.05 significance level. This was based on the assumption that the standard deviation of the response variable was 0.03, as had been found in our preliminary data (unpublished). This allowed for not only comparison between T1D and healthy controls, but also for detection of differences between sexes. In this small-scale study, missing data were managed using a variable-specific exclusion approach. Participants with missing values for a particular variable were excluded only from analyses involving that variable, while remaining included in analyses where their data were complete. Statistical analyses were performed with IBM SPSS 23.0. The Shapiro–Wilk test was used to test variables for normal distribution and independent sample t-test was used for comparison between CWD and healthy controls, except for WBC, which was not normally distributed and therefore compared by using nonparametric Mann-Whitney-U test. Groups were also stratified by sex and compared using the same statistical methods. Variables are presented as mean ± SD (median (IQR) for WBC), and correlations between variables were investigated by Pearson's rho correlation coefficients, a two-sided p-value <0.05 was considered statistically significant. Collected glycaemic and metabolic markers, time in tight range (TITR), time in range (TIR), coefficient of variation (CV), standard deviation from mean glucose (SD), mean glucose, HbA1c, and longitudinal HbA1c as well as BP, BP z-score, blood lipid levels, cystatin C, and WBC, were included in the correlation analysis, and analysed in relation to our primary outcomes the vascular wall measures IT, MT, and IMT, of the radial, DP, and carotid arteries, respectively. Linear univariate regression was conducted on variables identified as significant in the correlation analysis. Backwards multivariable regression analysis was performed to investigate determinants for all vascular wall measures, on the total study population and on the type 1 diabetes group separately. Multivariable regression models were built from the significant predictors in univariate regression and other pre-known important variables that may affect the cardiovascular status, such as BP- and BMI z-score, age and sex, in regression analysis on total study population, type 1 diabetes diagnosis was also included as an independent variable. Presented values are beta coefficients (Standard Error (SE)), R and R^2^ values from the first and final significant model obtained from the regression analysis.

### Role of the funding source

The funders of the study had no role in study design, data collection, data analysis, data interpretation, or writing of the report. Authors had full access to the study data and FD was responsible for the decision of submission of the manuscript.

## Results

### Demographic data

**Fig. 1:**
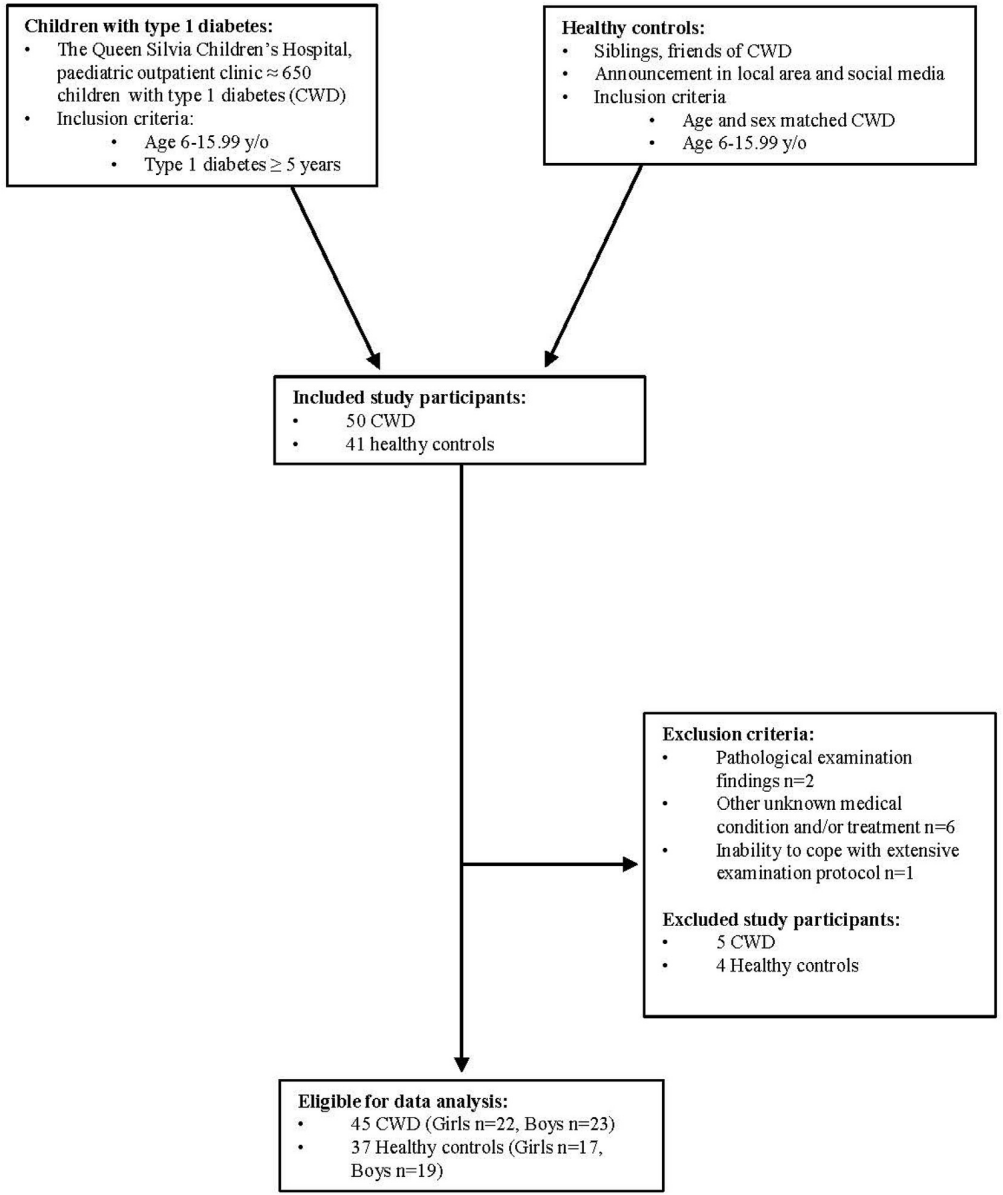
Flow chart of participant enrolment, inclusion and exclusion criteria, and number of enrolled study participants as well as number of girls and boys, respectively.

50 CWD and 41 healthy controls were included in the study, five CWD and four healthy controls met the exclusion criteria, and their examination results were removed from data analysis. Results from the remaining 45 CWD, 22 girls and 23 boys, and 37 healthy controls, 18 girls and 19 boys, were included in the data analysis (Fig. 1). No differences in age or body size were found between CWD and healthy controls (Table 1). All CWD had a CGM device, 5/45 CWD were treated with multiple injections using an insulin pen, and 40/45 used an insulin pump. Mean HbA1c in CWD was 6.6 ± 0.6% (48.1 ± 6.0 mmol/mol), longitudinal HbA1c was 6.9 ± 2.8% (51.4 ± 6.6 mmol/mol) (Table 1).

**Table 1:** Demographic data, comparison between study participants with type 1 diabetes and healthy controls.

	Type 1 diabetes n = 45	HC n = 37	p-value
Demographic data			
Age (years)	12.0 ± 2.3	11.3 ± 2.5	0.19
Sex (girls (boys)) (n/%)	22/49 (23/51)	18/49 (19/51)	–
Diagnosis duration (years)	7.22 (5.25, 11.46)	–	–
Height (cm)	156.5 ± 15.0	152.3 ± 16.3	0.23
Weight (kg)	48.6 ± 14.4	44.7 ± 13.7	0.22
Waist (cm)	69.2 ± 7.4	67.9 ± 9.3	0.47
Hip (cm)	85.2 ± 9.5	82.2 ± 11.0	0.20
BMI z-score (SD)	0.35 ± 0.77	0.29 ± 0.89	0.73
SBP (mmHg)	106 ± 7	103 ± 8	0.12
DBP (mmHg)	66 ± 4	63 ± 5	0.0053
Pulse pressure (mmHg)	40 ± 6	41 ± 4	0.70
SBP z-score	0.50 ± 0.23	0.45 ± 0.23	0.38
DBP z-score	0.59 ± 0.17	0.50 ± 0.18	0.019
Blood samples			
HbA1c% (mmol/mol)	6.55 ± 0.6 (48.1 ± 6.0)	5.00 ± 0.21 (31.09 ± 2.22)	<0.0001
Longitudinal HbA1c% (mmol/mol)	6.9 ± 2.8 (51.4 ± 6.6)	–	–
WBC	5.5 (4.0, 10.7)	5.8 (4.1, 12.6)	0.45
Cystatin C	0.89 ± 0.14	0.86 ± 0.10	0.26
eGFR (ml/min)	104.28 ± 16.89	111.24 ± 16.42	0.080
Cholesterol	4.09 ± 0.56	4.02 ± 0.63	0.61
Triglycerides	0.72 ± 0.56	1.05 ± 0.47	0.0005
HDL	1.50 ± 0.23	1.28 ± 0.26	0.0003
LDL	2.31 ± 0.50	2.47 ± 0.56	0.19

Comparison between groups using independent sample t-test, and nonparametric Mann-Whitney-U test when not normally distributed. Values presented as mean ± SD and median (range) when not normally distributed. Abbreviations: CWD- children with diabetes, HC- Healthy controls, BMI- Body mass index, SBP- Systolic blood pressure, DBP- Diastolic blood pressure, WBC- White cell count, HDL- High-density lipoprotein, LDL- low density lipoprotein, HbA1c- Haemoglobin A1c, Longitudinal HbA1c- Mean HbA1c from every 3rd month after type 1 diabetes diagnosis.

### Blood pressure

Office DBP and DBP z-score were significantly higher in CWD as compared to healthy controls (p = 0.0053, and p = 0.019, respectively) (Table 1). Office DBP also differed in separate analysis of boys and girls, with a significantly higher DBP in CWD irrespective of sex (p = 0.054 for boys, and p = 0.043 for girls, respectively) (Supplementary Table S1).

### Biochemistry

CWD showed significantly lower triglycerides and higher HDL levels than healthy controls (p = 0.0005, and p = 0.0003, respectively) (Table 1). The increase in HDL in CWD persisted in separate analysis of boys and girls (p = 0.0026, and p = 0.043, respectively) (Supplementary Table S1).

### Vascular ultrasound

**Fig. 2:**
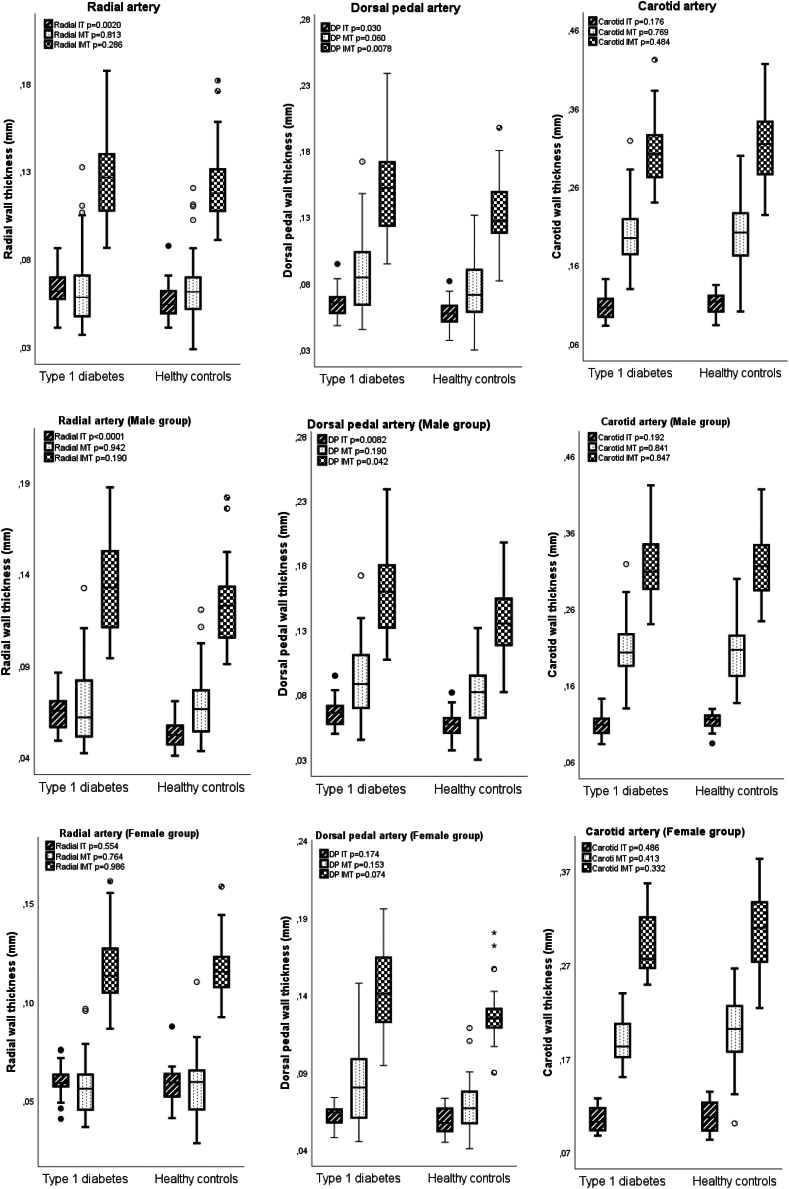
Boxplots with results from UHFUS measurements in total-, male- , and female study population. Independent sample t-test for comparison between groups, p-values presented for each vascular measure. Abbreviations: IT- intima thickness, MT- Media thickness, IMT- Intima- Media thickness, DP- Dorsal pedal. Pattern code: Stripes = IT, dots = MT, squares = IMT.

Radial IT, and DP IT and IMT was increased in CWD, as compared to healthy controls (p = 0.0020, p = 0.0030, and p = 0.0078, respectively) (Table 2). Boys with type 1 diabetes showed higher radial IT, and DP IT and IMT, compared to healthy boys (p < 0.0001, p = 0.0082, and p = 0.042, respectively). Boys with type 1 diabetes also displayed larger diameter in all examined arteries, compared to girls with type 1 diabetes (Radial: p = 0.0089, DP: p = 0.029, and Carotid: p = 0.029) (Fig. 2, Supplementary Table S2).

**Table 2:** Continuous glucose monitoring (CGM) metrics from last 14 days before study occasion.

CGM metrics in CWD	CWD n = 31	Boys n = 17	Girls n = 14	p-value
Longitudinal HbA1c (%, mmol/mol)	6.9 ± 2.8 51.4 ± 6.6	6.9 ± 2,8 51.0 ± 6.9	6.9 ± 2,7 51.8 ± 6.3	0.67
TIR (%) (3.9–10.0 mmol/l, 70–180 mg/dL)	63.6 ± 9.7	63.0 ± 10.4	64.4 ± 9.1	0.59
TITR (%) (3.9–7.8 mmol/l, 70–140 mg/dL)	41.5 ± 9.9	42.4 ± 9.2	40.4 ± 4.6	0.71
TBR (%) (<3.9,70 mg/dL)	4.0 ± 2.7	5.2 ± 2.7	2.6 ± 1.9	0.0055
SD	3.4 ± 0.6	3.6 ± 0.7	3,1 ± 0.49	0.28
CV (%)	39.8 ± 5.15	41.9 ± 4.7	37.4 ± 4.6	0.012
Mean glucose (mmol/l)	8.6 ± 1.0	8.4 ± 1.0	8.9 ± 1.0	0.25

These data where available in 31 of the children with type 1 diabetes, 22 boys and 14 girls. Comparison between girls and boys with type 1 diabetes with independent sample t-test. Values presented as mean ± SD. Abbreviations: CWD- children with diabetes, TIR- Time in range (3.9–10.0 mmol/l, 70–180 mg/dL), TTTR- Time in tight range (3.9–7.8 mmol/l, 70–140 mg/dL), SD- Standard deviation from mean glucose, CV- Coefficient of variation, TBR- Time below range (<3.9,70 mg/dL).

Comparison between boys and girls with type 1 diabetes.

In healthy controls, boys showed a larger DP diameter than girls (p = 0.0036), and a lower DP IT/diameter ratio (p = 0.011) (Supplementary Table S2).

### Correlations

#### General correlations

Vascular diameter correlated with height, weight, BMI, waist- and hip circumference and age in CWD and healthy controls (r = 0.314–0.522, p = 0.0002–0.040). MT and IMT across all examined arteries were also generally correlated with age, height, weight, BMI, waist- and hip circumference in both study populations (r = 0.331–0.594, p < 0.0001–0.038).

#### Carotid artery

**Fig. 3:**
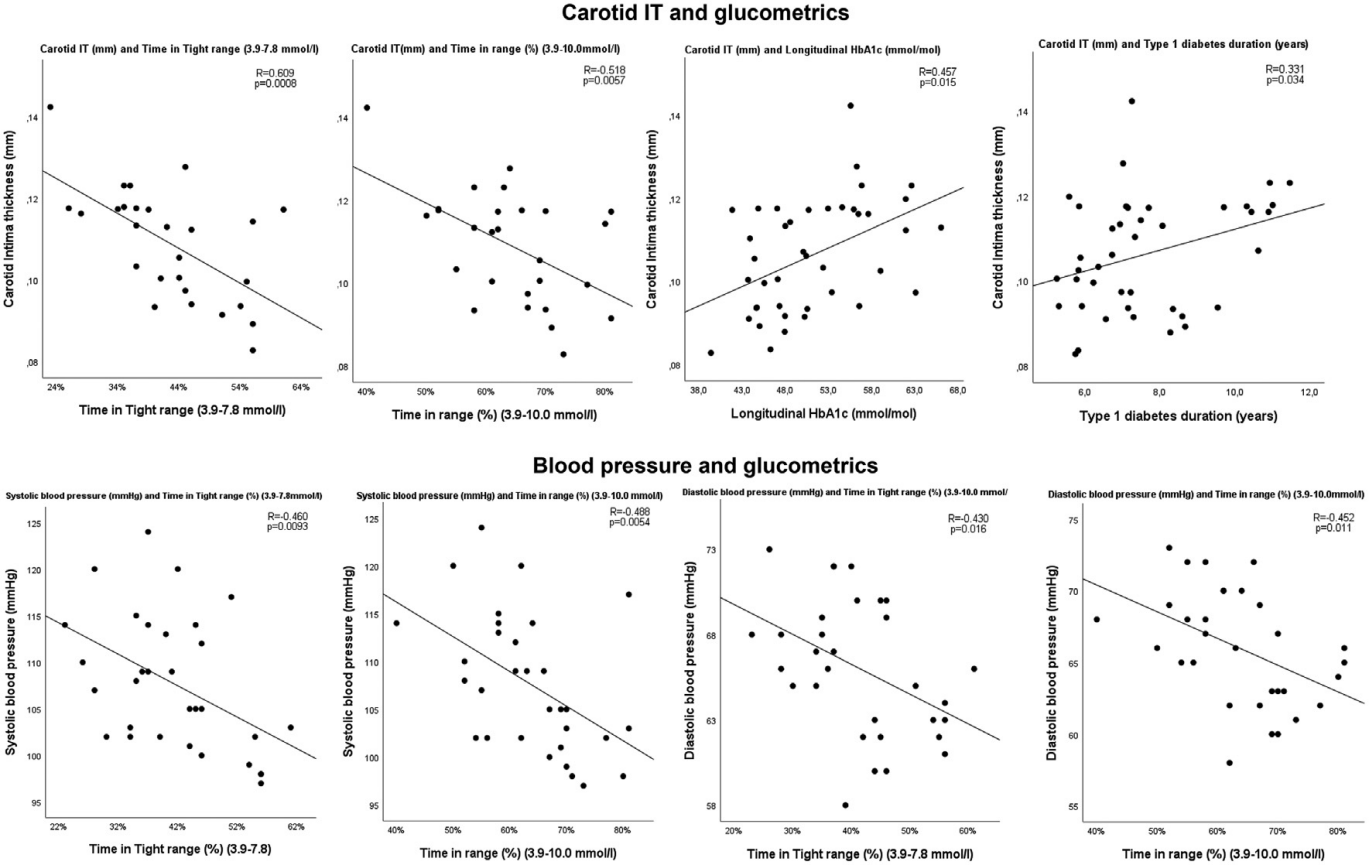
Important correlations for carotid IT and blood pressure. Statistical correlation by Pearson's rho, two-sided p-value <0.05 was considered statistically significant, correlation coefficient (R), and p-values are presented in each plotted correlation. Abbreviations: Time in range- % of time in blood sugar range 3.9–10.0 mmol/mol (70–180 mg/dL), Time in Tight range- % of time in the blood sugar range 3.9–7.8 mmol/mol (70–140 mg/dL), IT- Intima thickness, Longitudinal HbA1c- Mean HbA1c from every 3rd month after type 1 diabetes diagnosis.

In CWD, carotid IT and IT/diameter ratio showed negative correlations with TITR and TIR (IT: r = −0.609, p = 0.0008, and r = −0.518, p = 0.0057 IT/diameter: r = 0.428, p = 0.026 and r = −0.403, p = 0.037, respectively) (Fig. 3), and carotid IT showed positive correlations with mean glucose SD (r = 0.477, p = 0.012, and r = 0.425, p = 0.027, respectively). Carotid IT also correlated with type 1 diabetes duration, HbA1c and longitudinal HbA1c (r = 0.331, p = 0.034, r = 0.381, p = 0.015, and r = 0.457, p = 0.0027, respectively) (Fig. 3), and carotid IT/diameter ratio with HbA1c and longitudinal HbA1c (r = 0.334, p = 0.033, and r = 0.390, p = 0.013, respectively).

Carotid MT and IMT showed positive correlations with SBP, pulse pressure, and cystatin c (MT: r = 0.314, p = 0.046, r = 0.329, p = 0.035, and r = 0.338, p = 0.035 IMT: r = 0.392, p = 0.011, r = 0.404, p = 0.0089, and r = 0.416, p = 0.0083, respectively) as well as negative correlations with estimated glomerular filtration rate (eGFR), (MT: r = −0.368, p = 0.030, and IMT: r = −0.442, p = 0.0079, respectively). Carotid MT/diameter ratio also showed a negative correlation with eGFR (r = −0.337, p = 0.048) in CWD.

#### Radial artery

In CWD, radial diameter showed a negative correlation with DBP z-score (r = −0.465, p = 0.0013) and radial MT was positively correlated with HbA1c, SBP and pulse pressure (r = 0.333, p = 0.027, r = 0.518, p = 0.0003, and r = 0.582, p < 0.00001, respectively). Radial IMT and MT/diameter ratio also showed correlations with SBP and pulse pressure (IMT: r = 0.526, p = 0.0002, and r = 0.631, p < 0.0001, and MT/diameter: r = 0.439, p = 0.0025, and r = 0.386, p = 0.0088, respectively). Radial IT/diameter ratio was negatively correlated with weight and BMI z-score (r = −0.405, p = 0.0058, and r = −0.368, p = 0.013, respectively).

#### Dorsal pedal artery

DP IT/diameter ratio correlated with HDL and eGFR in CWD (r = −0.356, p = 0.022, and r = 0.331, p = 0.042, respectively). In healthy controls, DP IT showed a positive correlation with cystatin C (r = 0.389, p = 0.025), DP IT/diameter ratio with total cholesterol (r = 0.347, p = 0.048) and DP MT with HbA1c (r = 0.419, p = 0.015).

#### Blood pressure

In CWD, office SBP and DBP negatively correlated with TITR and TIR (SBP: r = −0.460, p = 0.0093, and r = −0.488, p = 0.0054, DBP: r = −0.430, p = 0.016, and r = −0.452, p = 0.011, respectively) (Fig. 3). SBP positively correlated with HbA1c, longitudinal HbA1c, SD, and mean glucose (r = 317, p = 0.036, r = 0.316, p = 0.034, r = 0.522, p = 0.0026, and r = 0.467, p = 0.0081, respectively) and SBP z-score with SD and mean glucose (r = 0.448, p = 0.011, and r = 0.412, p = 0.021, respectively).

### Regression analysis

#### Univariate regression

In univariate regression analysis in CWD, carotid IT was associated with HbA1c, longitudinal HbA1c, TITR, TIR, SD, and mean glucose (r = 0.381, p = 0.015, r = 0.457, p = 0.0027, r = 0.609, p = 0.0008, r = 0.518, p = 0.0057, r = 0.425, p = 0.027, and r = 0.477, p = 0.012 respectively), and carotid IT/diameter ratio was associated with HbA1c, longitudinal HbA1c, TITR, and TIR (r = 0.390, p = 0.013, r = 0.328, p = 0.036, r = 0.428, p = 0.026, and r = 0.403, p = 0.037, respectively). Carotid MT was associated with cystatin C and eGFR (r = 0.338, p = 0.035, and r = 0.368, p = 0.030, respectively), radial MT was associated with HbA1c (r = 0.333, p = 0.027) and radial IMT was associated with longitudinal HbA1c (r = 0.314, p = 0.036).

#### Multivariable regression

##### Total study population

Backwards multivariable regression analyses were performed on all arterial wall measures, IT, MT, IMT, IT/diameter ratio, and MT/diameter ratio. Significant models are shown in detail in Supplementary Table S3. HbA1c, type 1 diabetes diagnosis, age, sex, BMI- SBP- and DBP z-score were used as independent variables. Type 1 diabetes diagnosis was one of the most, or the most, important determinant for IT and IT/diameter ratio across all examined arteries (r = 0.335–0.471, p = 0.0006–0.018). The final model for carotid MT included BMI z-score and age (r = 0.465, p = 0.0003), and for carotid IMT and MT/diameter ratio, only age was included as independent variable (r = 0.400, p = 0.0003, and r = 0.381, p = 0.0005, respectively). Analyses on radial MT and IMT resulted in final models with age, BMI z-score, HbA1c and type 1 diabetes diagnosis as independent variables (r = 0.589, p < 0.0001, and r = 0.577, p < 0.0001, respectively). The final model for DP MT included age, BMI z-score, and HbA1c whereas the final model for DP IMT included age, BMI z-score and type 1 diabetes diagnosis (r = 0.386, p = 0.0092 and r = 0.407, p = 0.0048) (Supplementary Table S3).

##### Children with type 1 diabetes

In backwards multivariable regression analyses, on vascular measures from CWD, TITR, CV, longitudinal HbA1c, BMI z-score, SBP- and DBP z-score, age, and sex were used as independent variables. Analysis for carotid IT resulted in a three-variable model including TITR, longitudinal HbA1c, and sex, where TITR and longitudinal HbA1c were separately significant (r = 0.743, p = 0.0003). The final model for carotid IT/diameter ratio included TITR, CV and sex, both TITR and sex were separately significant (r = 0.647, p = 0.0053). Similar analyses for carotid MT and IMT resulted in two univariate models including age (r = 0.440, p = 0.022, and r = 0.460, p = 0.016) (Supplementary Table S3).

Results from analysis on radial artery showed a final model for radial IT/diameter ratio including BMI-, DBP z-score, and sex, where BMI- and DBP z-score were separately significant (r = 0.656, p = 0.001). Final model for radial MT included TITR, SBP z-score and age, with SBP z-score and age separately significant (r = 0.691, p = 0.0014), final model for radial IMT included SBP-, DBP z-score and age, with SBP z-score and age separately significant (r = 0.713, p = 0.0003), and final model for radial MT/diameter ratio included age (r = 0.445, p = 0.041) (Supplementary Table S3).

No significant determinants for DP wall thickness in neither uni- nor multivariable regression models were found in the CWD.

## Discussion

Regardless of comparably good glycaemic control, absence of modifiable CV risk factors and an even more favourable lipid profile compared to healthy controls (Fig. 1, Fig. 3), CWD in this study display signs of structural changes to peripheral arteries. These changes involve predominately the intima, and especially the DP artery, with significantly increased DP IT, IMT and borderline significantly increased DP MT in CWD (Table 2, Fig. 2). In regression analysis, type 1 diabetes diagnosis was one of, or the most important, determinant for IT across all examined arteries, and carotid IT showed strong associations with glucometrics.

**Table 3:** Ultra-high frequency ultrasound measurements in subjects with type 1 diabetes compared to healthy controls.

	CWD n = 45	Healthy controls n = 37	p-value
Radial artery			
Diameter (mm)	1.740 ± 0.307	1.676 ± 0.255	0.32
IT (mm)	0.062 ± 0.009	0.056 ± 0.010	0.0020
MT (mm)	0.064 ± 0.022	0.065 ± 0.021	0.81
IMT (mm)	0.126 ± 0.026	0.121 ± 0.021	0.29
IT/diameter	0.037 ± 0.006	0.034 ± 0.008	0.10
MT/diameter	0.037 ± 0.011	0.039 ± 0.013	0.35
Dorsal pedal artery			
Diameter (mm)	1.420 ± 0.403	1.319 ± 0.357	0.25
IT (mm)	0.064 ± 0.010	0.058 ± 0.010	0.0030
MT (mm)	0.087 ± 0.029	0.075 ± 0.023	0.060
IMT (mm)	0.151 ± 0.032	0.133 ± 0.026	0.0078
IT/diameter	0.049 ± 0.017	0.048 ± 0.018	0.71
MT/diameter	0.064 ± 0.019	0.058 ± 0.014	0.18
Carotid artery			
Diameter (mm)	5.988 ± 0.391	6.034 ± 0.374	0.60
IT (mm)	0.106 ± 0.013	0.111 ± 0.013	0.18
MT (mm)	0.198 ± 0.036	0.201 ± 0.043	0.77
IMT (mm)	0.305 ± 0.040	0.312 ± 0.046	0.48
IT/diameter	0.018 ± 0.002	0.018 ± 0.002	0.31
MT/diameter	0.033 ± 0.007	0.033 ± 0.007	0.99

Comparison between groups with independent sample t-test. Abbreviations: CWD- Children with diabetes, IT- Intima thickness, MT- media thickness, IMT- Intima- Media thickness.

To the best of our knowledge, this study is the first to show the association between IT and hyperglycaemia, implying that striving for normoglycemia is perhaps the most crucial CV-preventive action in CWD. We also show the applicability of using UHFUS for examining small peripheral arteries and the value of differentiation of IT and MT for detection of minor and possibly earlier vascular changes in CWD.

Increased cIMT has been previously described in CWD with higher HbA1c than the children in this study.^15^^,^^16^^,^^21^^,^^22^ CIMT has been associated with several of the glucometrics, such as TIR, time below range (TBR), and glucose variability.^11^ However, cIMT constitutes a composite measure of IT and MT and is therefore less likely to be affected in children reaching glycaemic target goals. The largest part of cIMT is the media, which is more likely to be affected by high blood pressure, another important risk factor for atherosclerotic changes. This likely makes cIMT a less sensitive maker than the separate measures of IT and MT used in this study. The comparably good glycaemic control and low prevalence of traditional CV risk factors as compared to CWD in other studies,^7^^,^^8^^,^^23^ and the fact that carotid IT is a very small percentage of cIMT are feasible explanations for detection of changes only in the more peripheral, radial and DP arteries in our study cohort.

DP IT has previously been shown to increase earlier than radial- and carotid IT in healthy individuals by age, likely explained by more pronounced BP exposure compared to other peripheral arteries.^19^ In our study, we found elevated DBP and DBP z-score and corresponding changes in the DP artery in CWD (Table 1, Table 2). We also found an increased radial IT in CWD, not persisting when adjusting for diameter. However, type 1 diabetes was the most important determinant for radial IT in multivariable regression, which increases the likelihood that this is mediated by type 1 diabetes (Table 2, Supplementary Table S3).

Differentiation between IT and MT allowed for detailed pathophysiological considerations. Firstly, type 1 diabetes was the most important determinant for IT (radial, DP, and carotid) in multivariable regression, stronger than age, sex, BMI- and BP z-score (Supplementary Table S3). Secondly, carotid IT as well as carotid IT/diameter ratio showed strong associations with several of the glucometrics, TITR being the strongest (Fig. 3). In regression analysis in CWD, TITR was the most important determinant for carotid IT and IT/diameter ratio. This suggests that hyperglycaemia in type 1 diabetes has a direct impact on IT, this is to the best of our knowledge the first time this association has been described. Since children in general, even with type 1 diabetes, have less traditional CV risk factors, and since we did not include any children with other medical treatment than insulin, and the CWD in this study did not display elevated LDL-levels or hypertension, we can assume that the vasculature is predominately impacted by the dysglycemia. We therefore consider our findings in line with, and emphasising, strict glycaemic control as perhaps the most important CV preventive action in type 1 diabetes.^2^^,^^3^

From several previous studies we know the CV preventive effects of intensive insulin treatment and striving for normoglycemia in type 1 diabetes.^24^^,^^25^ In a recent study including 34,263 individuals with type 1 diabetes, peripheral vascular disease decreased over time with increasing numbers of patients reaching glycaemic target goals.^26^ However, even with this established knowledge of a link between hyperglycaemia and early CVD complications, achieving normalised HbA1c seems to be a difficult challenge in young individuals with type 1 diabetes.^27^

Vascular diameter was larger in all three arteries in boys compared to girls with type 1 diabetes and in the DP artery in healthy controls (Table 2). This is consistent with earlier findings, where increased radial diameter, IT, MT, and IMT have been observed in healthy boys compared to girls.^28^ Diameter, MT and IMT were strongly associated with anthropometrics, suggesting that these measures are more associated with age and body size than with type 1 diabetes. This is consistent with results from the ALSPAC study, where slight changes to the media are interpreted as a response to the change in body composition naturally taking place, rather than a sign of subclinical atherosclerosis.^29^ However, some findings from this study complicates these theories. Radial MT was associated with HbA1c in CWD. In multivariable regression, HbA1c and/or type 1 diabetes diagnosis were important independent variables for radial MT and IMT as well as for DP MT and IMT. In addition, carotid MT was associated with BP-z-score and kidney function in CWD. These findings may indicate a parallel process where type 1 diabetes also affect MT, mediated by BP and kidney function. Explanations for increased MT in type 1 diabetes include microvascular changes in the kidneys, contributing to diabetic kidney disease. Associations between BP z-score and glucometrics was also found, in line with earlier studies where elevated BP have been associated with higher HbA1c levels, and traditional CVD risk-factors seem to enhance the vascular damaging effect of hyperglycemia.^30^

Strengths to this study are continuity of staff during study inclusion, minimising bias due to missing data or misinterpretations, the age-range in our study population, the relatively large study population and the age- and sex-matched healthy controls. Available CGM data and homogenous diabetes treatment with CGM combined with insulin pump therapy in most of the CWD, and access to longitudinal glycaemic information from NDR are also considered strengths.

Our CWD study population constitute a socioeconomically homogenous group with no prevalence of traditional CV risk factors, similar life circumstances, and are matched to children from a similar life environment. This provides an isolated examination of type 1 diabetes mediated CV impact in these CWD, excluding as many confounding factors as possible.

An important limitation is that the study was conducted during the COVID-19 pandemic. We do not yet know what long-term impact COVID-19 has on the cardiovascular system, and we had no possibility to exclude all with a confirmed infection before baseline due to the widespread infection. Another limitation is that we did not collect total daily insulin dose from CWD, this would have been an interesting factor to analyse in relation to the morphological vascular assessment.

In conclusion, even these children, with what is considered very well-regulated type 1 diabetes and without other CV risk-factors, display changes in peripheral arteries. Hyperglycaemia seems to play a significant role in increasing IT. Small and possibly earlier changes than previously described seem to be detectible in more peripheral arteries, predominately in DP, through differentiation between IT and MT. Our results also indicate a parallel process involving hypertension and impact on kidney function probably contributing to increased MT.

Hence, striving for normoglycaemia is essential in cardiovascular prevention in type 1 diabetes already in children, in addition to early monitoring and possibly pharmaceutical treatment for hypertension and hyperlipidaemia. When working with populations like this study cohort of CWD with good glycaemic control, sensitive methods for detecting and assessing vascular impact over time are essential. Additional studies with similar set-up are needed to further confirm these results, as well as future evaluations of earlier and more aggressive treatment strategies.

## Contributors

FD and GF were responsible for the conceptualisation of the study. EB and LM included and examined patients. EB measured UHFUS examinations, compiled data, performed statistical analysis, wrote the first draft of the manuscript. FS, GF, and FD revised and edited and contributed to all parts of the manuscript. All authors approved the final version of the manuscript. FD and EB had full access to the data throughout the study, and FD is the guarantor of this study with responsibility of data integrity and accuracy of data analysis.

## Data sharing statement

Anonymised underlying data are available upon request by contacting the corresponding author.

## Declaration of interests

GF was partially financed by Vinnova, the Swedish Agency for Innovation Systems. She was also invited speaker at the “International society for paediatric ad adolescent diabetes” (ISPAD) annual meeting, Lisbon, 2024 and, head of ISPAD special interest group for diabetes in school, 2022–2024. The rest of the co-authors have no conflicts of interest to declare.
