# Supplementary material for: Investigating the presence and detectability of structural peripheral arterial changes in children with well-regulated type 1 diabetes versus healthy controls using ultra-high frequency ultrasound: a single-centre cross-sectional and case-control study
Source: eClinicalMedicine. 2025 Feb 13;81:103097. doi: 10.1016/j.eclinm.2025.103097 (PMC11872503; doi:10.1016/j.eclinm.2025.103097)
Supplement: Supplementary Tables S1–S3 [file mmc1.pdf]

Supplementary table 1. Demographic data, comparison between study participants with type 1 diabetes and healthy controls stratified by sex.

Supplementary table 1. Demographic data and blood samples CWD compared to healthy controls, groups stratified by sex						
	**Girls CWD n=22**	**Girls HC n=18**	**p-value**	**Boys CWD n=23**	**Boys HC n=19**	**p-value**
**Demographics**						
**Age (years)**	11.9±2.4	11.4±2.6	0.51	12.1±2.4	11.3±2.4	0.25
**Diabetes duration (years)**	7.8 ± 1.7		--	7.6±2.0		--
**Height (cm)**	153.6±14.3	151.3±15.3	0.63	159.3±15.4	153.3±17.7	0.24
**Weight (kg)**	46.6±14.6	43.1±12.0	0.43	50.5±14.3	46.2±15.2	0.36
**Waist (cm)**	68.2±8.5	66.6±9.0	0.58	70.2±6.3	69.0±9.7	0.64
**Hip (cm)**	84.4±9.8	82.3±10.1	0.52	85.8±9.4	82.1±12.0	0.26
**BMI z-score (SD)**	0.27±0.81	0.14±0.85	0.62	0.42±0.73	0.43±0.92	0.97
**SBP (mmHg)**	104±5	103±7	0.64	108±8	103±9	0.12
**DBP (mmHg)**	66±4	63±5	0.043	65±4	62±6	0.054
**Pulse pressure (mmHg)**	38±3	40±4	0.083	43±7	42±5	0.56
**SBP z-score**	0.49±0.25	0.45±0.25	0.64	0.50±0.21	0.45±0.21	0.43
**DBP z-score**	0.60±0.17	0.53±0.16	0.16	0.58±0.17	0.47±0.19	0.066
**Blood samples**						
**HbA1c %(mmol/mol)**	6.51±0.55 (47.61±5.99)	5.06±0.18 (31.78±2.02)	<0.0001	6.60±0.56 (48.57±6.07)	4.92±0.21 (30.31±2.24)	<0.0001
**Longitudinal HbA1c% (mmol/mol)**	6.9±2,7 (51.8± 6.3)	--	--	6.9±2,8 (51.0±6.9)	--	--
**WBC**	6.3 (4.5, 10.7)	5.6 (4.1, 12.6)	0.031	5.0 (4.0, 6.8)	6.1 (4.5, 11.0)	0.0020
**Cystatin C**	0.90±0.15	0.87±0.12	0.34	0.88±0.12	0.84±0.09	0.48
**eGFR (ml/min)**	101.47±16.93	108.94±1754	0.21	106.95±16.84	113.81±15.20	0.20
**Cholesterol**	4.11±0.70	3.88±0.62	0.53	4.08±0.38	4.18±0.62	0.30
**Triglycerides**	0.65±0.28	1.05±0.45	0.064	0.79±0.26	1.04±0.52	0.0017
**HDL**	1.54±0.27	1.27±0.26	0.043	1.45±0.17	1.30±0.27	0.0026
**LDL**	2.29±0.64	2.34±0.61	0.039	2.34±0.34	2.63±0.48	0.79

Comparison between groups using independent sample t-test. Values presented as mean ± SD. Abbreviations: CWD- children with diabetes, HC- Healthy controls, BMI – Body mass index, SBP – Systolic blood pressure, DBP – Diastolic blood pressure, WBC- White cell count, HDL – High density lipoprotein, LDL – low density lipoprotein, HbA1c- Haemoglobin A1c, Longitudinal HbA1c - Mean HbA1c from every 3rd month after type 1 diabetes diagnosis

Supplementary table 2: Ultra high frequency ultrasound measurements in subjects with type 1 diabetes compared to healthy controls, groups stratified by sex

**Supplementary table 2. UHFUS measurements, groups stratified by sex CWD compared to healthy controls**						
**Examined artery (mm)**	**Girls CWD n=22**	**Girls HCn=18**	**p-value**	**Boys CWD n=23**	**Boys HCn=19**	**p-value**
**Radial artery**						
**Diameter (mm)**	1.620±0.300	1.632±0.196	0.88	1.854±0.274	1.717±0.301	0.13
**IT (mm)**	0.060±0.0081	0.058±0.011	0.55	0.065±0.010	0.053±0.009	<0.0001
**MT (mm)**	0.058±0.017	0.059±0.019	0.76	0.070±0.025	0.071±0.021	0.92
**IMT (mm)**	0.118±0.021	0.118±0.016	0.97	0.135±0.027	0.124±0.026	0.19
**IT/ diameter**	0.038±0.007	0.036±0.009	0.52	0.035±0.004	0.032±0,007	0.052
**MT/ diameter**	0.036±0.010	0.037±0.014	0.80	0.038±0.012	0.042±0.012	0.31
**Dorsal pedal artery**						
**Diameter (mm)**	1.288±0.387	1.143±0.285	0.20	1.551±0.383	1.477±0.347	0.52
**IT (mm)**	0.062±0.007	0.059±0.009	0.74	0.066±0.011	0.057±0.010	0.0082
**MT (mm)**	0.082±0.026	0.070±0.021	0.15	0.092±0.032	0.080±0.025	0.19
**IMT (mm)**	0.144±0.027	0.129±0.023	0.074	0.158±0.035	0.136±0.029	0.042
**IT/ diameter**	0.053±0.019	0.056±0.018	0.17	0.045±0.013	0.041±0.015	0.34
**MT/ diameter**	0.067±0.022	0.063±0.0156	0.51	0.060±0.017	0.054±0.012	0.20
**Carotid artery**						
**Diameter (mm)**	5.843±0.378	5.990±0.220	0.16	6.112±0.366	6.084±0.498	0.84
**IT (mm)**	0.105±0.013	0.109±0.015	0.49	0.107±0.014	0.113±0.011	0.19
**MT (mm)**	0.188±0.027	0.198±0.044	0.41	0.207±0.042	0.204±0.044	0.84
**IMT (mm)**	0.294±0.034	0.307±0.046	0.33	0.314±0.042	0.317±0.047	0.85
**IT/diameter**	0.018±0.003	0.018±0.002	0.98	0.018±0.002	0.019±0.002	0.13
**MT/diameter**	0.032±0.005	0.033±0.007	0.73	0.034±0.008	0.033±0.007	0.83

Comparison between groups with independent sample t-test. Abbreviations: CWD-Children with type 1 diabetes, HC-Healthy controls, IT-Intima thickness, MT- media thickness, IMT- Intima- Media thickness, UHFUS- Ultra high frequency ultrasound

Supplementary table 3: Backwards multivariable regression analysis for total study population and type 1 diabetes group, respectively.

Supplementary table 3. Backwards multivariable regression *on UHFUS measures total study population*

**Carotid IT**				
**Variables**	**Model 1**	**p-value**	**Model 2**	**p-value**
**HbA1c**	0.0008 (0.0003)	0.017	0.0008 (0.0003)	0.013
**Type 1 diabetes**	0.0187 (0.0063)	0.004	0.0185 (0.0063)	0.0047
**Sex**	−0.0039 (0.0031)	0.22		
**BMI z-score**	−0.0025 (0.0019)	0.19		
**SBP z-score**	0.0077 (0.0068)	0.27		
**DBP z-score**				
**Age**				
**Intercept**	0.0509 (0.0224)		0.0476 (0.0217)	
**R-value**	0.404		0.334	
**R^2-value**	0.163		0.112	
**Model p-value**	0.037		0.018	
**Carotid MT**				
**Variables**	**Model 1**	**p-value**	**Model 2**	**p-value**
**HbA1c**	−0.001 (0.001)	0.46		
**Type 1 diabetes**	−0.006 (0.018)	0.75		
**Sex**	−0.006 (0.009)	0.51		
**BMI z-score**	0.010 (0.006)	0.076	0.010 (0.005)	0.073
**SBP z-score**	0.011 (0.024)	0.65		
**DBP z-score**	−0.017 (0.031)	0.57		
**Age**	0.008 (0.002)	0.0004	0.007 (0.002)	0.0002
**Intercept**	0.159 (0.066)		0.114 (0.021)	
**R-value**	0.489		0.465	
**R^2-value**	0.239		0.216	
**Model p-value**	0.013		0.0003	
**Carotid IMT**				
**Variables**	**Model 1**	**p-value**	**Model 2**	**p-value**
**HbA1c**	0.0001 (0.001)	0.95		
**Type 1 diabetes**	0.013 (0.020)	0.52		
**Sex**	-0.010 (0.010)	0.32		
**BMI z-score**	0.007 (0.006)	0.24		
**SBP z-score**	0.017 (0.026)	0.53		
**DBP z-score**	-0.011 (0.034)	0.75		
**Age**	0.007 (0.002)	0.002	0.007 (0.002)	0.0005
**Intercept**	0.206 (0.073)		0.223 (0.024)	
**R-value**	0.469		0.400	
**R^2-value**	0.220		0.160	
**Model p-value**	0.023		0.0005	
**Carotid IT/diameter**				
**Variables**	**Model 1**	**p-value**	**Model 2**	**p-value**
**HbA1c**	0.0002 (0.0001)	0.0029	0.0002 (0.0001)	0.0024
**Type 1 diabetes**	0.004 (0.001)	0.0009	0.003 (0.001)	0.0014
**Sex**	-0.0003 (0.0005)	0.55		
**BMI z-score**	-0.001 (0.0003	0.0011	-0.001 (0.0003)	0.0028
**SBP z-score**	0.0001 (0.001)	0.97		
**DBP z-score**	0.002 (0.002)	0.22		
**Age**	-0.0001 (0.0001)	0.47		
**Intercept**	0.006 (0.004)		0.007 (0.003)	
**R-value**	0.511		0.471	
**R^2-value**	0.261		0.221	
**Model p-value**	0.0061		0.0007	
**Carotid MT/diameter**				
**Variables**	**Model 1**	**p-value**	**Model 2**	**p-value**
**HbA1c**	−0.0001 (0.0002)	**0.73**		
**Type 1 diabetes**	−0.0003 (0.0033)	0.90		
**Sex**	−0.0003 (0.0016)	0.88		
**BMI z-score**	0.0006 (0.0010)	0.55		
**SBP z-score**				
**DBP z-score**				
**Age**	0.0011 (0.0003)	0.0029	0.001 (0.0003)	0.0010
**Intercept**	0.0231 (0.0116)		0.020 (0.004)	
**R-value**	0.392		0.381	
**R^2-value**	0.154		0.145	
**Model p-value**	0.050		0.0010	

Radial IT				
**Variables**	**Model 1**	**p-value**	**Model 2**	**p-value**
**HbA1c**	−0.0001 (0.0002)	0.66		
**Type 1 diabetes**	−0.0093 (0.0046)	0.048	-0.007 (0.002)	0.0028
**Sex**				
**BMI z-score**	−0.0007 (0.0014)	0.61		
**SBP z-score**	0.0056 (0.0058)	0.34		
**DBP z-score**	−0.0110 (0.0077)	0.16		
**Age**				
**Intercept**	0.0807 (0.0160)		0.069 (0.003)	
**R-value**	0.147		0.335	
**R^2-value**	0.384		0.113	
**Model p-value**	0.041		0.0028	

Radial MT				
**Variables**	**Model 1**	**p-value**	**Model 2**	**p-value**
**HbA1c**	0.001 (0.0004)	0.043	0.001 (0.0004)	0.036
**Type 1 diabetes**	0.017 (0.008)	0.048	0.019 (0.008)	0.028
**Sex**	-0.010 (0.004)	0.013	-0.011 (0.004)	0.0078
**BMI z-score**	-0.0004 (0.003)	0.88		
**SBP z-score**	0.012 (0.011)	0.30		
**DBP z-score**	-0.021 (0.014)	0.14		
**Age**	0.004 (0.001)	<0.0001	0.004 (0.001)	<0.0001
**Intercept**	-0.023 (0.030)		-0.030 (0.029)	
**R-value**	0.617		0.598	
**R^2-value**	0.381		0.358	
**Model p-value**	<0.0001		<0.0001	

Radial IMT				
**Variables**	**Model 1**	**p-value**	**Model 2**	**p-value**
**HbA1c**	0.001 (0.0005)	0.12	0.0005 (0.0002)	0.062
**Type 1 diabetes**	0.007 (0.009)	0.44	-0.010 (0.004)	0.023
**Sex**	-0.010 (0.005)	0.028	-0.022 (0.013)	0.099
**BMI z-score**	-0.001 (0.003)	0.70		
**SBP z-score**	0.018 (0.012)	0.15		
**DBP z-score**	-0.032 (0.016)	0.046		
**Age**	0.004 (0.001)	0.0001	0.004 (0.001)	0.0002
**Intercept**	0.056 (0.034)		0.086 (0.016)	
**R-value**	0.604		0.577	
**R^2-value**	0.364		0.335	
**Model p-value**	<0.0001		<0.0001	

Radial IT/diameter				
**Variables**	**Model 1**	**p-value**	**Model 2**	**p-value**
**HbA1c**	−0.0001 (0.0002)	0.53		
**Type 1 diabetes**	−0.0044 (0.0031)	0.17	−0.0032 (0.0015)	0.034
**Sex**	0.0031 (0.0015)	0.040	0.0034 (0.0015)	0.026
**BMI z-score**	−0.0027 (0.0010)	0.0065	−0.0025 (0.0009)	0.0090
**SBP z-score**	−0.0025 (0.0041)	0.54		
**DBP z-score**	0.0072 (0.0053)	0.18		
**Age**	−0.0006 (0.0003)	0.091	−0.0007 (0.0003)	0.039
**Intercept**	0.0462 (0.0112)		0.0434 (0.0051)	
**R-value**	0.511		0.487	
**R^2-value**	0.261		0.237	
**Model p-value**	0.0030		0.0006	

Radial MT/diameter				
**Variables**	**Model 1**	**p-value**	**Model 2**	**p-value**
**HbA1c**	0.0005 (0.0003)	0.097	0.0005 (0.0003)	0.096
**Type 1 diabetes**	0.0109 (0.0054)	0.048	0.0105 (0.0053)	0.051
**Sex**	−0.0029 (0.0026)	0.27		
**BMI z-score**	−0.0032 (0.0016)	0.055	−0.0029 (0.0016)	0.071
**SBP z-score**	−0.0019 (0.0071)	0.79		
**DBP z-score**	0.0027 (0.0091)	0.76		
**Age**	0.0016 (0.0006)	0.010	0.0016 (0.0005)	0.0048
**Intercept**	−0.0109 (0.0193)		−0.0140 (0.0181)	
**R-value**	0.457		0.441	
**R^2-value**	0.209		0.194	
**Model p-value**	0.019		0.0034	

DP IT				
**Variables**	**Model 1**	**p-value**	**Model 2**	**p-value**
**HbA1c**	−0.0001 (0.0002)	0.61		

**Type 1 diabetes**	−0.0101 (0.0048)	0.037	-0.007 (0.002)	0.0032
**Sex**				
**BMI z-score**				
**SBP z-score**	0.0067 (0.0063)	0.29		
**DBP z-score**	-0.0128 (0.0079)	0.11		
**Age**	−0.0002 (0.0005)	0.71		
**Intercept**	0.0874 (0.0168)		0.071 (0.003)	
**R-value**	0.392		0.336	
**R^2-value**	0.154		0.113	
**Model p-value**	0.038		0.0032	
**DP MT**				
**Variables**	**Model 1**	**p-value**	**Model 2**	**p-value**
**HbA1c**	0.0005 (0.001)	0.45	0.001 (0.0003)	0.056
**Type 1 diabetes**	-0.002 (0.012)	0.87		
**Sex**	-0.008 (0.006)	0.20		
**BMI z-score**	-0.007 (0.004)	0.077	-0.006 (0.004)	0.085
**SBP z-score**	0.023 (0.016)	0.16		
**DBP z-score**	-0.017 (0.021)	0.43		
**Age**	0.003 (0.001)	0.030	0.003 (0.001)	0.056
**Intercept**	0.040 (0.044)		0.030 (0.018)	
**R-value**	0.446		0.386	
**R^2-value**	0.199		0.149	
**Model p-value**	0.031		0.0092	
**DP IMT**				
**Variables**	**Model 1**	**p-value**	**Model 2**	**p-value**
**HbA1c**	0.0004 (0.001)	0.61		
**Type 1 diabetes**	-0.012 (0.014)	0.39	-0.017 (0.007)	0.012
**Sex**	-0.008 (0.007)	0.21		
**BMI z-score**	-0.007 (0.004)	0.089	-0.007 (0.004)	0.10
**SBP z-score**	0.030 (0.018)	0.10		
**DBP z-score**	-0.029 (0.023)	0.22		
**Age**	0.003 (0.002)	0.065	0.002 (0.001)	0.086
**Intercept**	0.128 (0.049)		0.141 (0.021)	
**R-value**	0.480		0.407	
**R^2-value**	0.231		0.165	
**Model p-value**	0.011		0.0048	
**DP IT/diameter**				
**Variables**	**Model 1**	**p-value**	**Model 2**	**p-value**
**HbA1c**	−0.0003 (0.0004)	0.49		
**Type 1 diabetes**	−0.0074 (0.0080)	0.36		
**Sex**	0.0110 (0.0038)	0.005	0.0109 (0.0037)	0.004
**BMI z-score**				
**SBP z-score**	0.0044 (0.0105)	0.68		
**DBP z-score**	−0.0029 (0.0133)	0.83		
**Age**	−0.0018 (0.0009)	0.048	−0.0020 (0.0008)	0.017
**Intercept**	0.0759 (0.0286)		0.0558 (0.0111)	
**R-value**	0.427		0.413	
**R^2-value**	0.183		0.170	
**Model p-value**	0.028		0.0012	
**DP MT/diameter**				
**Variables**	**Model 1**	**p-value**	**Model 2**	**p-value**
**HbA1c**				
**Type 1 diabetes**	−0.0040 (0.0040)	0.33		
**Sex**	0.0082 (0.0039)	0.041	0.0082 (0.0039)	0.040
**BMI z-score**	−0.0051 (0.0024)	0.039	−0.0049 (0.0024)	0.046
**SBP z-score**	0.0099 (0.0091)	0.28		
**DBP z-score**				
**Age**	0.0006 (0.00090)	0.72		
**Intercept**	0.0446 (0.0163)		0.0512 (0.0063)	
**R-value**	0.392		0.344	
**R^2-value**	0.154		0.119	
**Model p-value**	0.038		0.011	
**Children with type 1 diabetes**				
**Carotid IT**				
**Variables**	**Model 1**	**p-value**	**Model 2**	**p-value**
**TITR**	−0.0007 (0.0003)	0.013	−0,0007 (0.0002)	0.0042
**CV**	0.0004 (0.0005)	0.38		
**Longitudinal HbA1c**	0.0011 (0.0004)	0.016	0.0008 (0.0003)	0.018
**BMI z-score**	−0.0020 (0.0030)	0.52		

**SBP z-score**	−0.0099 (0.0137)	0.48		
**DBP z-score**	0.0030 (0.0161)	0.85		
**Age**	−0.0015 (0.0011)	0.21		
**Sex**	0.0072 (0.0047)	0.14	0.0068 (0.0038)	0.088
**Intercept**	0.0766 (0.0389)		0.0875 (0.0218)	
**R-value**	0.799		0.743	
**R^2-value**	0.638		0.552	
**Model p-value**	0.0067		0.0003	
**Carotid MT**				
**Variables**	**Model 1**	**p-value**	**Model 2**	**p-value**
**TITR**				
**CV**				
**Longitudinal HbA1c**				
**BMI z-score**	0.0087 (0.0079)	0.28		
**SBP z-score**				
**DBP z-score**				
**Age**	0.0065 (0.0026)	0.020	0.0064 (0.0026)	0.022
**Sex**				
**Intercept (B)**	0.1258 (0.0320)		0.1297 (0.0319)	
**R-value**	0.482		0.440	
**R^2-value**	0.233		0.194	
**Model p-value**	0.042		0.0229	
**Carotid IMT**				
**Variables**	**Model 1**	**p-value**	**Model 2**	**p-value**
**TITR**				
**CV**				
**Longitudinal HbA1c**				
**BMI z-score**				
**SBP z-score**				
**DBP z-score**	0.0501 (0.0442)	0.27		
**Age**	0.0082 (0.0029)	0.0091	0.0071 (0.0027)	0.016
**Sex**				
**Intercept**	0.1869 (0.0509)		0.2308 (0.0332)	
**R-value**	0.502		0.460	
**R^2-value**	0.252		0.212	
**Model p-value**	0.031		0.016	
**Carotid IT/diameter**				
**Variables**	**Model 2**	**p-value**	**Model 3**	**p-value**
**TITR**	−0.0001 (0.0001)	0.057	−0.0001(0.00004)	0.032
**CV**	0.0001 (0.0001)	0.21	0.0001(0.0001)	0.098
**Longitudinal HbA1c**	0.0001(0.0001)	0.14		
**BMI z-score**	−0.001 (0.001)	0.40		
**SBP z-score**	−0.002 (0.003)	0.44		
**DBP z-score**				
**Age**	−0.0002 (0.0002)	0.33		
**Sex**	0.002 (0.001)	0.024	0.003(0.001)	0.0063
**Intercept**	0.0123 (0.008)		0.0126 (0.005)	
**R-value**	0.708		0.647	
**R^2-value**	0.501		0.419	
**Model p-value**	0.039		0.0053	
**Radial MT**				
**Variables**	**Model 1**	**p-value**	**Model 2**	**p-value**
**TITR**	0.001 (0.0004)	0.19	0.001 (0.0004)	0.080
**CV**	−0.001 (0.001)	0.41		
**Longitudinal HbA1c**	0.001 (0.001)	0.36		
**BMI z-score**	−0.001 (0.005)	0.82		
**SBP z-score**	0.046 (0.023)	0.059	0.042 (0.018)	0.027
**DBP z-score**	−0.030 (0.029)	0.31		
**Age**	0.006 (0.002)	0.0038	0.0083 (0.002)	<0.0001
**Sex**	−0.008 (0.008)	0.33		
**Intercept**	−0.035 (0.068)		−0.087 (0.038)	
**R-value**	0.743		0.691	
**R^2-value**	0.553		0.477	
**Model p-value**	0.011		0.0005	
**Radial IMT**				
**Variables**	**Model 1**	**p-value**	**Model 2**	**p-value**
**TITR**	0.0006 (0.0005)	0.19		
**CV**	−0.0004 (0.0009)	0.66		
**Longitudinal HbA1c**	0.0008 (0.0007)	0.27		

**BMI z-score**	-0.0032 (0.0052)	0.54		
**SBP z-score**	0.0524 (0.0240)	0.039	0.0539(0.0200)	0.012
**DBP z-score**	−0.0358(0.0304)	0.25	−0.0536 (0.0283)	0.069
**Age**	0.0071 (0.0021)	0.0027	0.0068 (0.0016)	0.0003
**Sex**	−0.0088 (0.0088)	0.33		
**Intercept**	0.0014 (0.0711)		0.0514 (0.0284)	
**R-value**	0.766		0.713	
**R^2-value**	0.587		0.509	
**Model p-value**	0.0053		0.0003	
**Radial IT/D**				
**Variables**	**Model 1**	**p-value**	**Model 2**	**p-value**
**TITR**	0.00003 (0.0001)	0.79		
**CV**	0.0002 (0.0002)	0.50		
**Longitudinal HbA1c**	−0.0001 (0.0002)	0.78		
**BMI z-score**	−0.0036 (0.0014)	0.018	−0.0041 (0.0012)	0.0021
**SBP z-score**				
**DBP z-score**	0.01111 (0.0073)	0.14	0.0129 (0.0058)	0.034
**Age**	−0.0002 (0.0005)	0.76		
**Sex**	0.0046 (0.0023)	0.059	0.0037 (0.0018)	0.056
**Intercept**	0.0222 (0.0192)		0.0258 (0.0041)	
**R-value**	0.675		0.656	
**R^2-value**	0.456		0.431	
**Model p-value**	0.031		0.0014	
**Radial MT/D**				
**Variables**	**Model 1**	**p-value**	**Model 2**	**p-value**
**TITR**	0.0003 (0.0002)	0.21		
**CV**				
**Longitudinal HbA1c**				
**BMI z-score**	−0.0031 (0.0025)	0.23		
**SBP z-score**	0.0175 (0.0106)	0.11		
**DBP z-score**				
**Age**	0.0032 (0.0010)	0.0033	0.0023 (0.0008)	0.012
**Sex**				
**Intercept**	−0.0216 (0.0223)		0.0105 (0.0104)	
**R-value**	0.556		0.445	
**R^2-value**	0.309		0.198	
**Model p-value**	0.041		0.012	

First and last significant model from backwards multivariable regression is presented, independent variables included in the analysis are listed under every vascular measure. Values presented: beta coefficient, (standard error (SE)), R, R^2, Intercept. Abbreviations: IT- intima thickness, MT- media thickness, IMT- intima- media thickness, IT/D- intima/ diameter ratio, MT/D- media diameter ratio, TITR- Time in tight range, CV- coefficient of variation, BMI- body mass index, SBP- systolic blood pressure, DBP- diastolic blood pressure.
